# Neighbourhood socioeconomic deprivation and health-related quality of life: A multilevel analysis

**DOI:** 10.1371/journal.pone.0188736

**Published:** 2017-12-13

**Authors:** Vânia Rocha, Ana Isabel Ribeiro, Milton Severo, Henrique Barros, Sílvia Fraga

**Affiliations:** 1 EPIUnit—Instituto de Saúde Pública, Universidade do Porto, Porto, Portugal; 2 Departamento de Ciências da Saúde Pública e Forenses e Educação Médica, Faculdade de Medicina, Universidade do Porto, Porto, Portugal; Scientific Institute of Public Health (WIV-ISP), BELGIUM

## Abstract

**Objective:**

To assess the relationship between socioeconomic deprivation and health-related quality of life in urban neighbourhoods, using a multilevel approach.

**Methods:**

Of the population-based cohort EPIPorto, 1154 georeferenced participants completed the 36-Item Short-Form Health Survey. Neighbourhood socioeconomic deprivation classes were estimated using latent-class analysis. Multilevel models measured clustering and contextual effects of neighbourhood deprivation on physical and mental HRQoL.

**Results:**

Residents from the least deprived neighbourhoods had higher physical HRQoL. Neighbourhood socioeconomic deprivation together with individual-level variables (age, gender and education) and health-related factors (smoking, alcohol consumption, sedentariness and chronic diseases) explained 98% of the total between-neighbourhood variance. Neighbourhood socioeconomic deprivation was significantly associated with physical health when comparing least and most deprived neighbourhoods (class 2—beta coefficient: -0.60; 95% confidence interval:-1.76;-0.56; class 3 –beta coefficient: -2.28; 95% confidence interval:-3.96;-0.60), and as neighbourhood deprivation increases, a decrease in all values of physical health dimensions (physical functioning, role physical, bodily pain and general health) was also observed. Regarding the mental health dimension, no neighbourhood clustering or contextual effects were found. However, as neighbourhood deprivation increases, the values of vitality and role emotional dimensions significantly decreased.

**Conclusion:**

Neighbourhood socioeconomic deprivation is associated with HRQoL, affecting particularly physical health. This study suggests that to improve HRQoL, people and places should be targeted simultaneously.

## 1. Introduction

Health-related quality of life (HRQoL) is a measure of perceived health status, and has become an important endpoint to monitor the population’s health, because it captures subjective assessments of both physical and mental health [1, 2]. The broad, multidimensional and subjective nature of HRQoL reflects numerous factors, such as age, health-related behaviours, social support and the presence of medical conditions [3–5]. The socioeconomic factors, for example, education and occupation, have been associated with HRQoL over the life-course, and individuals from lower socioeconomic positions seem to experience worse quality of life than those at the top [3, 6].

Studying the “local context” association with individual health and well-being has been identified as a key research priority, as neighbourhoods have been considered the arena where interpersonal interactions take place, values and culture are formed, and consumption habits and daily routines are established [3], which may ultimately influence health and health behaviours. Exploring specifically HRQoL has the potential to provide a holistic perspective on health status, as HRQol includes several mental and physical aspects [1, 2]. Nevertheless, studying this relationship requires controlling for multiple variables that might confound the association between neighbourhood deprivation and HRQoL, for example health-related behaviours. Thereby, smoking, excessive alcohol consumption, and physical inactivity were more prevalent in the most deprived areas and were associated with higher levels of chronic diseases, which might influence HRQoL [7].

Neighbourhood deprivation was also related with older people’s subjective satisfaction with physical function and environment, independently of education, social class, cognitive ability and number of diseases [8]. Additionally, consistent decreasing trends of self-rated health and quality of life with the increase of neighbourhood deprivation were reported [6, 9–13]. High levels of unemployment, income inequality, poor housing conditions and overcrowding in the neighbourhoods were also associated with poor health perceptions [14–16]. Yet, some studies found a weak influence of the neighbourhood-level variables when compared to the more powerful influences of individual level factors [17, 18], and some showed that neighbourhood socioeconomic deprivation did not exert any influence over HRQoL [19].

Overall, controversy still exists about the association of neighbourhood socioeconomic deprivation with HRQoL, and numerous pathways might explain this association. On the one hand, neighbourhood physical environment is to a great extent conditioned by the socioeconomic structure of the neighbourhood, as least deprived neighbourhoods usually attract beneficial features as opposed to the most deprived neighbourhoods, which tend to concentrate more detrimental physical exposures due to an underinvestment in these areas [3]. Detrimental physical environments, characterized by lack of different facilities (recreational, healthcare, community services), pollution, absence of green spaces, poor housing conditions or noise might directly affect population’s health or shape health-related behaviours [20]. On the other hand, the socioeconomic environment of the neighbourhood can determine social norms, values and traditions, which might influence health-related behaviours and residents’ social and economic trajectories [21]. And, the social composition of the neighbourhood might directly affect mental well-being [22], as feelings of inferiority, stigma and lack of social support, disorder and crime are found to be more common in the most deprived places [23].

It is also important to mention that most research on this topic has been conducted in Anglo-Saxon countries, making inferences to other places difficult because neighbourhood associations might be context specific [12, 16, 21, 24]. More recently, research has also been conducted in other countries [4, 25], but specifically in the Portuguese context there are no studies about the association between neighbourhood deprivation and HRQoL. Yet, addressing this issue seems particularly relevant as, despite the relatively small territorial extent, Portugal is a country with high levels of social inequalities [26].

From a public health perspective, it is crucial to identify if there is an association between neighbourhood socioeconomic deprivation and HRQoL. If so, interventions need to be also targeted at improving the physical and social environment. Indeed, these kind of neighbourhood-level interventions are thought to be ‘equigenic’ [27], as they might improve residents’ quality of life, but, more importantly, they might effectively reduce inequalities in health [28]. Therefore, the aim of this study was to evaluate the association between socioeconomic deprivation in urban neighbourhoods and HRQoL, using a multilevel approach.

## 2. Methods

### 2.1 Study design and participants

This study was conducted using data from the EPIPorto cohort study previously described in detail [29]. Briefly, between 1999 and 2003, a representative sample of community dwellers of Porto was assembled. Households were selected by random digit dialling of landline telephones and within each household, a permanent resident aged 18 years or more was selected by simple random sampling; refusals were not replaced. A total of 2485 individuals composed the EPIPorto final sample, with a response proportion of 70% [29]. The last 1325 participants was consecutively invited to self-complete the 36-Item Short Form Health Survey (SF-36). From those 1325 individuals, 464 had missing information on the variables included in the analysis, leading to a final sample of 1155 participants.

### 2.2 Study area

Porto is a municipality located in the northwest of Continental Portugal and has approximately 215,000 inhabitants distributed across 41.7km^2^. It is the second largest metropolitan area of Portugal, near the Atlantic coast, along the Douro River estuary, and is an industrial and port town with roughly 1.3 million inhabitants. Regarding socioeconomic status, 50% of the Porto population lives in medium deprived areas. The spatial distribution of the areas by socioeconomic status (SES) follows a demarcated pattern–areas with similar levels of deprivation tend to be close to each other–revealing a high degree of socio-spatial. Porto also presents a compact urban design (relatively high residential density with mixed land uses) [30].

### 2.3 Measures and procedures

#### 2.3.1 Participants’ characteristics

A structured questionnaire to assess participants’ sociodemographic characteristics, lifestyles and chronic diseases was administered by trained interviewers during face-to-face interviews. Participants’ age was collected as completed years and classified in three categories, 18–34, 35–64 and 65 years or older. Education was recorded as completed years of schooling and classified in 3 categories: 4 years or less, 5–11, and more than 11. Participants were classified as current smokers, including both daily and occasional smokers, or other (non-smoker and former smoker). Alcohol consumption was estimated by asking participants about the consumption of different types of alcoholic beverages. Two categories of excessive alcohol consumption were defined according to the cut points 15.0 grams per day (g/day) for women and 30.0g/day for men, considering the American Heart Association recommendations [31].

Sedentariness was evaluated using a questionnaire exploring type of occupation and the frequency of household and leisure-time activities over the past 12 months [32]. For each group of leisure-time physical activities (light, moderate, vigorous) a metabolic equivalent (MET) value was assigned[32]. Participants were considered to be sedentary if they were classified in the lowest sex-specific third of daily leisure or exercise energy expenditure. The cut-off values were 237 and 270 METs.min/day for women and men, respectively.

All diagnosis of diseases that require regular medical care, such as asthma, diabetes or cardiovascular diseases, were recorded.

#### 2.3.2 Health-related quality of life assessment

The 36-Item Short Form Health Survey is a generic, self-administered questionnaire designed to capture two main domains: physical and mental health [33].

Physical health was assessed with four dimensions that characterized Physical Functioning (i.e., limitations in physical activities because of health problems), Role Physical (limitations in usual role activities because of physical health problems), Bodily Pain (the intensity of pain and the effect of pain on normal work), and General Health Perceptions (limitations in social activities because of physical or emotional problems).

Mental health was assessed with four dimensions that characterized Vitality (energy and fatigue), Social Functioning (the extent to which physical health or emotional problems interfere with normal social activities), Role Emotional (limitations in usual role activities because of emotional problems) and General Mental Health (psychological distress and well-being) [33].

This scale was validated for the Portuguese population [34] and its psychometric validity was also established [35]. Severo and colleagues [35] assessed the SF-36 internal consistency using the Cronbach's alpha and obtained an alpha of 0.82 and 0.87 for physical and mental health domains, respectively. This study [35] also reported that the two domains explained 70.4% of the variability in HRQoL and argued that the use of these two domains as summary measures allows the characterisation of HRQoL for clinical and research purposes. For our sample, which does not fully match the sample of the previous study [35], we also calculated the Cronbach's alpha, which was 0.81 and 0.87 for physical and mental health domains respectively, showing again a good internal consistency.

The SF-36 consists of eight scaled scores that are the weighted sums of the questions in their dimension. Each scale is directly transformed into a 0–100 scale on the assumption that each question carries equal weight. Therefore, the scores for each domain and respective dimensions range from 0 to 100 and higher scores represent a better HRQoL [33]. We calculated a summary score for each domain, physical and mental, following previous recommendations [33, 36].

#### 2.3.3 Neighbourhood socioeconomic deprivation classes

Neighbourhood of residence was established with the census block where individuals resided [37]. In Porto, in 2001, there were 2064 census blocks [38], and neighbourhood socioeconomic deprivation was computed for 1662 census blocks; the remaining 402 had 10 or less inhabitants, which prevented us to compute a robust socioeconomic deprivation measure for these areas [39]. Participants were georeferenced using ArcGIS Online World Geocoding Service and Google Maps. Then, point-in-polygon operations allowed us to determine each participant census block and the corresponding socioeconomic deprivation class [40]. The classification of neighbourhoods was built upon 47 variables available from the 2001 Census at the census block level, the most recent census evaluation at the time. The final classification included 11 variables: proportion of retired individuals, proportion of families with a person aged 15 years or less, aging index, illiteracy proportion, proportion of subjects with higher education, proportion of subjects with lower occupation, unemployment rate, mean expenditure on housing (owner occupied housing), mean expenditure on housing (rented housing), attractiveness (proportion of residents that resided in another territorial unit or country 5 years before) and proportion of buildings with reparation needs [39] (for detailed information on the neighbourhoods’ classification process see “S1 Text”). To create a summary measure that captured neighbourhood socioeconomic deprivation, latent class models were run to identify census blocks with similar characteristics [40]. The number of neighbourhoods’ classes was defined according to the Bayesian information criterion, the Akaike information criterion, entropy and interpretability and 3 discrete classes were defined. Class 1 (least deprived) accounted for 23% of the census blocks, composed of younger and highly educated populations; housing conditions were good, housing expenditure was high and unemployment rates were low. Class 2 (medium deprived) accounted for 47% of the census blocks, composed of intermediate proportions of damaged buildings, and intermediate levels of attractiveness and housing expenditure. Finally, class 3 (most deprived) accounted for 30% of the census blocks, characterized by a medium ageing index, low levels of education, attractiveness and housing expenditures, and high unemployment rates.

### 2.4 Statistical analysis

First, one-way anova was used to compare the mean scores of HRQoL domains and dimensions by neighbourhood socioeconomic deprivation classes. A multilevel linear regression analysis was also used considering a two-level hierarchical data structure, in which individuals were nested in neighbourhoods. Thus, from the 1662 neighbourhoods 223 were analysed, which included at least 3 participants, because multi-level designs are very sensitive to the number of observations by nesting unit [41]. We also repeated the analysis including neighbourhoods with at least 10 participants and the results were similar (results not shown), therefore we kept the first.

We estimated the association of neighbourhood deprivation and each outcome, by including a fixed effect slope for neighbourhood socioeconomic deprivation, computing beta coefficients (B) and the respective 95% confidence intervals (95%CI). Then, between-neighbourhood differences in the quality of life were assessed (Model1). Model 2 resulted from Model 1 plus the addition of neighbourhood socioeconomic deprivation class. Then, age, gender and education were added to the model (Model 3), aiming to control for plausible individual-level confounders. Previous evidence [5, 12, 16] suggested that people from most deprived neighbourhoods tended to be older and present less years of education. Finally, dichotomous variables about health-related behaviours–smoking, excessive alcohol intake, sedentariness and chronic diseases were also included in the model (Model 4), as it has been suggested that people from most deprived neighbourhoods are more likely to develop health-risk behaviours as smoking, drinking excessive alcohol and be sedentariness, leading to higher levels of chronic diseases [7]. These models allowed for the estimation of Intraclass Correlation Coefficient (ICC), a measure of clustering correlation that expresses the proportion of the total variance that is at the neighbourhood level. This modelling sequence also allowed for the estimation of the proportion of neighbourhood variance that could be explained by each set of variables using the Model 1 neighbourhood variance estimation as a reference. Analyses were performed using IBM SPSS 21.0 and R 2.14.1(‘nlme’ package). The level of significance considered was α = 0.05.

## 3. Results

Table 1 shows the characteristics of participants according to neighbourhood socioeconomic deprivation class. Participants residing in the least deprived areas were significantly younger (p<0.001), presented higher levels of education (p<0.001), were more frequently non-smokers (p = 0.001), not sedentary (p = 0.029) and had a lower prevalence of chronic diseases (p = 0.001).

**Table 1:** Participants’ characteristics according to the neighbourhood socioeconomic deprivation class.

n = 1154 [n (%)]	Total	Class 1 Least deprived (n = 361)	Class 2 (n = 623)	Class 3 Most deprived (n = 170)	Pearson chi-square statistic	Degrees of freedom	p-value
**Age**					35.35	4	**<0.001**
18–34 years	78	42 (11.6)	29 (4.7)	7 (4.1)			
35–64 years	818	267 (74.0)	427 (68.5)	124 (72.9)			
65 or more years	258	52 (14.4)	167 (26.8)	39 (22.9)			
**Gender**					1.31	2	0.519
Female	699	210 (58.2)	383 (61.5)	106 (62.4)			
Male	455	151 (41.8)	240 (38.5)	64 (37.6)			
**Education**					155.07	4	**<0.001**
4 years or less	401	58 (16.1)	234 (37.6)	109 (64.1)			
5 to 11 years	384	122 (33.8)	212 (34.0)	50 (29.4)			
12 or more years	369	181 (50.1)	177 (28.4)	11 (6.5)			
**Current smoking behaviour**					12.95	2	**0.002**
Smoker	263	105 (29.1)	119 (19.1)	39 (22.9)			
Non-smoker	891	256 (70.9)	504 (80.9)	131 (77.1)			
**Excessive alcohol intake**^**1**^					3.19	2	0.203
No	866	283 (78.4)	459 (73.7)	124 (72.9)			
Yes	288	78 (21.6)	164 (26.3)	46 (27.1)			
**Sedentariness**^**2**^					7.11	2	**0.029**
No	803	248 (68.7)	450 (72.2)	105 (61.8)			
Yes	351	113 (31.3)	173 (27.8)	65 (38.2)			
**Chronic Disease**					17.68	4	**0.001**
No	443	165 (45.7)	217 (34.8)	61 (35.9)			
Yes	710	196 (54.3)	406 (65.2)	108 (63.5)			

Legend

^1^Alcohol intake >15g/day for women and >30g/day for men.

^2^ Women and men were considered sedentary if they scored below 237 and 270 METs.min/day, respectively.

In bold statistically significant p-values.

Table 2 shows that mean scores of SF36 domains were significantly different between the three groups of socioeconomic neighbourhood deprivation, except for some dimensions of the mental health domain.

**Table 2:** Participants’ health-related quality of life according to neighbourhood socioeconomic deprivation classes.

n = 1154 (mean ±SD)	Class 1 Least deprived (n = 361)	Class 2 (n = 623)	Class 3 Most deprived (n = 170)	F statistic (One-way Anova)	Degrees of freedom	p-value
**Physical Health**	51.5±9.25	47.8±9.65	44.6±11.27			**<0.001**
Physical functioning	79.3±20.07	71.7±23.19	63.9±26.59	28.12	2	**<0.001**
Role physical	79.0±28.31	70.0±31.94	64.6±34.53	15.04	2	**<0.001**
Bodily pain	68.3±24.83	62.1±25.89	54.9±28.48	16.17	2	**<0.001**
General health perceptions	61.5±19.25	55.5±19.54	52.7±21.18	15.19	2	**<0.001**
**Mental Health**	49.9±10.25	49.4±10.57	49.9±10.04			0.745
Vitality	58.7±20.30	55.5±21.96	52.8±22.88	4.89	2	**0.008**
Social Functioning	76.2±23.19	73.8±25.05	72.9±25.07	1.47	2	0.230
Role emotional	78.3±27.68	70.7±32.29	66.7±33.81	10.14	2	**<0.001**
General mental health	66.4±21.78	64.0±23.50	62.3±24.01	2.20	2	0.111

Legend: SD: standard deviation.

In bold statistically significant p-values.

Table 3 shows the neighbourhood clustering and the contextual effect of the neighbourhood deprivation on the physical health of HRQoL. Physical health decreased significantly as neighbourhood socioeconomic deprivation increased, with a B of -3.68 (95%CI:-4.99;-2.37) in medium deprived neighbourhoods (class 2) and a B of -6.86 (95%CI:-8.70;-5.02) in the most deprived neighbourhood (class 3). The ICC revealed that 5.0% of the variance in the physical health of HRQoL is at neighbourhood-level and 71.8% of the total between-neighbourhood variance was explained by the neighbourhood socioeconomic deprivation class.

**Table 3:** Neighbourhood clustering and contextual effects of neighbourhood socioeconomic deprivation on physical health-related quality of life.

PHYSICAL HEALTH		Model 1*	Model 2*	Model 3*	Model 4*
**Fixed effects B(95%CI)**					
Class 1 –least deprived		—-	Ref.	Ref.	Ref.
Class 2		—-	**-3.68 (-4.99;-2.37)**	-0.66 (-1.83;0.51)	-0.60 (-1.76;0.56)
Class 3 –most deprived		—-	**-6.86 (-8.70;-5.02)**	**-2.41 (-4.10;-0.72)**	**-2.28 (-3.96;-0.60)**
**Random effects**					
Variance		5.0	1.41	1.75*10^−6^	0.10
Variance explained (%)ˠ		Ref.	71.8	99.9	98.0
ICC (%)		5.0	1.5	2.4*10^−6^	0.1
Residual (SD)		9.7	8.6	8.5	8.5
				
	PHYSICAL FUNCTIONING				
	**Fixed effects B(95%CI)**				
	Class 1 –least deprived	—-	Ref.	Ref.	Ref.
	Class 2	—-	**-7.54 (-10.51;-4.57)**	-0.89 (-3.65;1.87)	-0.84 (-3.59;1.91)
	Class 3 –most deprived	—-	**-15.42 (-19.59;-11.24)**	**-5.64 (-9.64;-1.64)**	**-5.46 (-9.44;-1.47)**
	**Random effects**				
	Variance	8.87	6.16*10^−6^	5.51*10^−6^	5.61*10^−6^
	Variance explained (%)ˠ	Ref.	99.9	99.9	99.9
	ICC (%)	1.6	1.2*10^−6^	1.3*10^−6^	1.4*10^−6^
	Residual (SD)	23.2	22.8	20.3	20.2
				
	ROLE PHYSICAL				
	**Fixed effects B(95%CI)**				
	Class 1 –least deprived	—-	Ref.	Ref.	Ref.
	Class 2	—-	**-8.99 (-13.11;-4.89)**	-1.65 (-5.63;2.33)	-1.47 (-5.48;2.54)
	Class 3 –most deprived	—-	**-14.41 (-20.19;-8.63)**	-3.20 (-8.96;2.57)	-2.90 (-8.70;2.89)
	**Random effects**				
	Variance	23.05	5.89	3.22	6.76
	Variance explained (%)ˠ	Ref.	74.5	86.0	70.7
	ICC (%)	2.3	0.6	0.4	0.8
	Residual (SD)	31.3	29.0	28.9	25.7
				
	BODILY PAIN				
	**Fixed effects B(95%CI)**				
	Class 1 –least deprived	—-	Ref.	Ref.	Ref.
	Class 2	—-	**-6.17 (-9.71;-2.63)**	-0.64 (-3.98;2.70)	-0.45 (-3.81;2.91)
	Class 3 –most deprived	—-	**-13.40 (-18.38;-8.42)**	-3.96 (-8.79;0.86)	-3.60 (-8.44;1.24)
	**Random effects**				
	Variance	31.18	18.45	13.16	18.62
	Variance explained (%)ˠ	Ref.	40.8	57.8	40.3
	ICC (%)	4.5	2.7	2.4	3.4
	Residual (SD)	25.6	23.3	23.0	19.6
				
	GENERAL HEALTH PERCEPTIONS				
	**Fixed effects B(95%CI)**	—-			
	Class 1 –least deprived	—-	Ref.	Ref.	Ref.
	Class 2	—-	**-5.99 (-8.63;-3.35)**	-1.76 (-4.33;0.82)	-1.64 (-4.18;0.89)
	Class 3 –most deprived	—-	**-8.83 (-12.55;-5.11)**	-2.60 (-6.32;1.12)	-2.18 (-5.85;1.48)
	**Random effects**				
	Variance	12.33	6.35	2.92	4.44
	Variance explained (%)ˠ	Ref.	48.5	76.3	63.9
	ICC (%)	3.1	1.6	0.8	1.3
	Residual (SD)	19.6	19.5	18.5	18.0

Legend: ICC, intraclass correlation coefficient. B = beta regression coefficients; SD: standard deviation; 95% CI: 95% confidence intervals

* Model 1: neighbourhood random effect only; Model 2: Model 1 plus fixed effects of neighbourhood socioeconomic deprivation class; Model 3: Model 2 plus age, gender and education; Model 4: Model 3 plus smoking, alcohol consumption, sedentariness and chronic diseases

ˠ Proportion of explained variance (%): corresponds to the proportion of between-neighbourhood variance that could be explained by measured neighbourhood variables, and individual-level confounders compared to Model 1, calculated as [1-(variance of the model/ variance of the reference model)]*100

Ref: Reference category. N = 1154.

The between-neighbourhood variance was further reduced with the inclusion of the individual-level variables of age, gender and education, and remained statistically significant when comparing least and most deprived neighbourhood (most deprived neighbourhoods: B of -2.41; 95%CI:-4.10;-0.72), but in medium deprived neighbourhoods lost significance (model 3). These variables together with the neighbourhood socioeconomic deprivation class virtually explained all the variability. In model 4, after adding smoking, alcohol consumption, sedentariness and chronic diseases, the variance explained was slightly lower, and the association between physical health and neighbourhood socioeconomic deprivation kept its significance, when comparing least and most deprived neighbourhoods, class 3 with a B of -2.28 (95%CI:-3.96;-0.60).

Considering the physical health dimensions (physical functioning, role physical, bodily pain and general health), we observed that as neighbourhood socioeconomic increases the HRQoL in these dimensions significantly decrease (model 2). Neighbourhood socioeconomic deprivation explained 99.9%, 74.5%, 40.8% and 48.5% of the variance in physical functioning, role physical, bodily pain and general health, respectively. Further, the between-neighbourhood variance was reduced with the inclusion of the individual-level variables of age, gender and education, with the variance explained being the same for physical functioning and increasing for role physical (86.0%), bodily pain (57.8%) and general health (76.3%) (model 3).

Table 4 showed no evidence of neighbourhood clustering or contextual effects on the mental health of HRQoL, except for the vitality and role emotional dimensions, which varied between neighbourhoods and were influenced by neighbourhood deprivation, as shown previously in the Table 2. The neighbourhood socioeconomic deprivation explained 22.2% of the total between-neighbourhood variance in mental health of HRQoL.

**Table 4:** Neighbourhood clustering and contextual effects of neighbourhood socioeconomic deprivation on mental health-related quality of life.

MENTAL HEALTH		Model 1*	Model 2*	Model 3*	Model 4*
**Fixed effects B (95%CI)**					
Class 1 –least deprived		—-	Ref.	Ref.	Ref.
Class 2		—-	-0.47(-1.82;0.88)	-0.14 (-1.52;1.23)	-0.18 (-1.55;1.20)
Class 3 –most deprived		—-	-0.01(-1.92;1.89)	0.89 (-1.11;2.88)	1.00 (-0.99;2.99)
**Random effects**					
Variance		0.18	0.14	0.46	0.37
Variance explained (%)ˠ		Ref.	22.2	155.6	105.6
ICC (%)		0.2	0.1	0.5	0.4
Residual (SD)		10.4	10.0	9.9	9.8
				
	VITALITY				
	**Fixed effects B (95%CI)**				
	Class 1 –least deprived	—-	Ref.	Ref.	Ref.
	Class 2	—-	**-3.30 (-6.22;-0.37)**	0.15 (-2.68;2.99)	0.12 (-2.69;2.94)
	Class 3 –most deprived	—-	**-5.77 (-9.90;-1.65)**	-0.12 (-4.22;3.97)	0.17 (-3.90;4.23)
	**Random effects**				
	Variance	14.02	11.30	7.31	7.57
	Variance explained (%)ˠ	Ref.	19.4	47.8	46.0
	ICC (%)	3.0	2.4	1.8	1.9
	Residual (SD)	21.3	21.3	20.0	19.8
				
	SOCIAL FUNCTIONING				
	**Fixed effects B (95%CI)**				
	Class 1 –least deprived	—-	Ref.	Ref.	Ref.
	Class 2	—-	-2.38 (-5.56;0.80)	-0.29 (-3.52;2.95)	-0.35 (-3.57;2.87)
	Class 3 –most deprived	—-	-3.31 (-7.78;1.16)	0.47 (-4.21;5.15)	0.72 (-3.94;5.38)
	**Random effects**				
	Variance	8.60*10^−6^	9.14*10^−6^	0.21	0.34
	Variance explained (%)ˠ	Ref.	6.28	2.4*10^6^	3.9*10^6^
	ICC (%)	1.4*10^−6^	1.5*10^−6^	0.03	0.06
	Residual (SD)	24.5	24.5	23.7	23.6
				
	ROLE EMOTIONAL				
	**Fixed effects B(95%CI)**				
	Class 1 –least deprived	—-	Ref.	Ref.	Ref.
	Class 2	—-	**-7.55 (-11.59;-3.50)**	-3.11 (-7.18;0.97)	-3.04 (-7.11;1.03)
	Class 3 –most deprived	—-	**-11.56 (-17.25;-5.87)**	-4.25 (-10.15;1.65)	-3.94 (-9.83;1.95)
	**Random effects**				
	Variance	9.66*10^−5^	1.27*10^−5^	1.52*10^−5^	1.44*10^−5^
	Variance explained (%)ˠ	Ref	86.9	84.3	85.1
	ICC (%)	9.8*10^−6^	1.3*10^−6^	1.7*10^−6^	1.6*10^−6^
	Residual (SD)	31.4	31.2	30.0	29.9
				
	GENERAL MENTAL HEALTH				
	**Fixed effects B (95%CI)**				
	Class 1 –least deprived	—-	Ref.	Ref.	Ref.
	Class 2	—-	-2.42 (-5.48;0.63)	0.23 (-2.82;3.28)	0.21 (-2.80;3.22)
	Class 3 –most deprived	—-	-4.26 (-8.56;0.04)	0.89 (-3.51;5.30)	1.25 (-3.11;5.60)
	**Random effects**				
	Variance	5.63	5.67	7.30	6.75
	Variance explained (%)ˠ	Ref.	0.7	29.7	19.9
	ICC (%)	1.06	1.1	1.5	1.5
	Residual (SD)	23.0	22.9	21.6	21.4

Legend: ICC, intraclass correlation coefficient. B = beta regression coefficients; SD: standard deviation; 95% CI: 95% confidence intervals.

* Model 1: neighbourhood random effect only; Model 2: Model 1 plus fixed effects of neighbourhood socioeconomic deprivation class; Model 3: Model 2 plus age, gender and education; Model 4: Model 3 plus smoking, alcohol consumption, sedentariness and chronic diseases.

ˠ Proportion of explained variance (%): corresponds to the proportion of between-neighbourhood variance that could be explained by measured neighbourhood variables, and individual-level confounders compared to Model 1, calculated as [1-(variance of the model/ variance of the reference model)]*100; Ref: Reference category. N = 1154.

Vitality significantly decreased as the neighbourhood socioeconomic class deprivation increased, with a B of -3.30 (95%CI:-6.22;-0.37) in medium deprived neighbourhoods (class 2) and a B of -5.77 (95%CI:-9.90;-1.65) in the most deprived neighbourhood (class 3). The ICC revealed that 3.0% of the variance in the vitality is at neighbourhood-level. Socioeconomic deprivation class explained 19.4% of the total between-neighbourhood variance in this dimension. Then, when the individual variables of age, gender and education were added, the variance explained raised to 47.8% and plus smoking, alcohol consumption, sedentariness and chronic diseases the variance explained was 46.0%.

Regarding the role emotional dimension, the beta coefficients significantly decreased as the neighbourhood socioeconomic class deprivation increased, with a B of -7.55 (95%CI:-11.59;-3.50) in medium deprived neighbourhoods and a B of -11.56 (95%CI:-17.25;-5.87) in the most deprived neighbourhood. Socioeconomic deprivation class explained 86.9% of the total between-neighbourhood variance. With the addition of the individual variables, age, gender and education, the variance explained was reduced to 84.3%; however, when fitting for smoking, alcohol consumption, sedentariness and chronic diseases the variance explained increased again to 85.1%.

## 4. Discussion

Neighbourhood socioeconomic deprivation was significantly associated with HRQoL, mainly with physical health. Residents from the most deprived neighbourhoods reported worse physical HRQoL than those from the least deprived neighbourhoods. This association remained even after adjustment for important individual sociodemographic and behaviour characteristics (model 3 and 4) and most of the significant differences in physical health were only maintained between people from the least and most deprived socioeconomic neighbourhoods, which suggests that the place where individuals live has influence over physical HRQoL, but differences were more evident between the extremes of socioeconomic deprivation spectrum, as previously reported [6, 42, 43].

Our findings were consistent with previous studies suggesting that living in more deprived neighbourhoods is associated with poorer physical health perception even after accounting for individual sociodemographic data, lifestyles and health status [6, 8, 9, 28, 44]. Some authors attribute these differences to the fact that people living in more deprived communities tend to feel badly in general and therefore are more likely to feel in poor health regardless their real physical state [11]. Specifically, regarding the physical health dimensions, there is also previous evidence [8] that neighbourhood deprivation may be associated with people’s satisfaction with their physical functioning (having good sleep and enough energy, getting around, being able to work and carry out daily activities), independently of the number of common adverse health conditions that people have. Corroborating our findings, a study also reported that residents from more deprived neighbourhoods experience higher levels of bodily pain, affecting normal work and a worse general health perceptions [45]. Although addressing a more generic indicator, self-rated health, several other studies observed that in more deprived neighbourhoods people tend to rate their own health status more poorly, which is also in accordance to our findings [11, 13].

Even though previous studies reported consistent associations between neighbourhood deprivation and mental health [12, 14, 46], in the present analysis, overall, we found no association (and no relevant between neighbourhood- variation). Similar findings were observed by other authors [8, 47] and lack of association with mental HRQoL could possibly derive from the use of census-based neighbourhood units, which might not be ideal to grasp neighbourhood-to-neighbourhood differences and the effect of neighbourhood deprivation on mental health. Additionally, it is important to notice that some of the studies that successfully demonstrated an association with mental health analysed the associations between mental health and income or income inequality [9, 14], rather than overall socioeconomic deprivation, suggesting that these indicators might be more appropriate to assess this relationship.

Nevertheless, we found that people from the most deprived areas had low scores on vitality and role emotional, meaning that those people were more likely to experience tiredness and emotional problems that interfere with work or daily activities. Indeed, there is previous evidence associating mental health aspects as stress, emotional problems and even depression to the characteristics of the neighbourhood [48]. According to Cutrona and colleagues [48], neighbourhood characteristics as poverty, deprivation and disorder greatly influence psychological processes, alongside the personal and family stressors, by increasing stress load, intensifying reactivity to negative life events, and damaging the quality of interpersonal relationships, leading to emotional problems. Thus, improving quality and reducing deprivation of neighbourhoods might have a positive effect not only in physical health but also in some aspects of mental health of its residents.

The modest association between neighbourhood deprivation and mental health provides some evidence that the association between neighbourhood deprivation and physical health might not be mediated by psychological mechanisms (stress, anxiety, feelings of inferiority). Indeed, van Jaarsvel [49], when exploring the pathways through which neighbourhood deprivation affects health, found that neighbourhood deprivation was more strongly related to behavioural than to psychosocial factors, whereas individual deprivation was strongly related to both. Therefore, we hypothesized that material exposures (poor housing and workplace conditions or lack of infrastructures and unemployment; and behaviours–smoking, poor diet, physical inactivity) are plausible mediators in the observed relation between neighbourhood socioeconomic deprivation and physical HRQoL, as the inclusion of these variables led to a reduction in the variability. However, in some cases this inclusion also led to a slight increase in the variability, which suggest that health-related behaviours could act as mediators but also as confounders in the relationship between socioeconomic deprivation and HRQoL.

Finally, our findings showed that HRQoL, specifically physical health, was influenced by both contextual and compositional factors. The contextual factors refer to the local physical and social environment and the compositional factors refer to the characteristics of the individuals living in specific places (demographics, socioeconomic status). These two factors were able to fully explain the variability in HRQoL, as previously reported [12], and indicates that the variability in physical health might depend on the physical and social environment of places where people live, but also on the demographic and socioeconomic characteristics of individuals.

Therefore, Public Health interventions aiming to improve quality of life might be more effective if targeting people and places simultaneously. Although our results did not allow identification of the particular matters that policy-makers should intervene in first, it is possible that infrastructural (e.g. provision of good quality open spaces, food environment and transportation) and social changes (e.g. reduction of crime, isolation and community participation barriers) at neighbourhood-level may have potential to improve the residents’ HRQoL.

### Strengths and limitations

The use of a reliable and validated measure of physical and mental health was a strong point of this study. The SF36 is widely used to compare HRQoL between different populations [11, 12, 33]. The use of a multilevel design to assess the influence of neighbourhood deprivation on HRQoL is widely recommended to differentiate contextual and compositional effects and have been pointed as the appropriate tool for examining area-level effects on individual health [20].

The definition of neighbourhood, grounded on administrative territorial divisions for analytical convenience, could be considered a limitation. The use of conceptual neighbourhoods (based on social networks and real-life routes) could be more appropriate [50], but unfortunately assessing past conceptual neighbourhoods was not possible. Our study only focused on physical and mental aspects of HRQoL, but we do recognize that quality of life involves other aspects of satisfaction with life, namely work life, communities and neighbourhoods. Probably the impact of neighbourhood socioeconomic deprivation would be larger if we had focused on those aspects.

Although our study was based in an urban setting, our findings may be applied to other urban contexts with similar patterns of socioeconomic deprivation. Additionally, the generalisability of the results are limited by the fact that only a part of the overall sample was asked to complete SF-36, and the missing data that led to the exclusion of 464 subjects. A sensitivity analysis was also performed to assess the differences between excluded and included participants, and included participants were younger and had higher level of education. Therefore, we could hypothesise that the effect would be even larger if we had included non-respondents in our analysis.

Regarding the selection of variables to be included in the deprivation index, poverty and income variables would be very valuable, but as the Portuguese census did not include these questions, and at individual-level, income and poverty were also not assessed in the EPIPorto cohort, we are not able of including them in the index construction, and this could have limited our results. Moreover, the inclusion of age in the deprivation index is debatable, but other socioeconomic deprivation indexes have included variables about the age structure of the population [51, 52].

Finally, due to data unavailability, we could not fully address the mechanisms through which neighbourhood deprivation impacts HRQoL (e.g. outdoor and indoor physical environments, social environment). It would be also interesting to explore the association of neighbourhood socioeconomic deprivation with HRQoL in the different socioeconomic groups.

## 5. Ethics statement

All procedures were in accordance with the ethical standards of the institutional and/or national research committee and with the 1964 Helsinki declaration and its later amendments or comparable ethical standards. The Hospital de São João Ethics Committee approved the study and participants provided written informed consent to participate in the study.

## Supporting information

S1 Text Detailed information on the process of neighbourhoods’ classification. (DOCX)
