# Supplementary material for: Neighbourhood socioeconomic deprivation and health-related quality of life: A multilevel analysis
Source: PLoS One. 2017 Dec 13;12(12):e0188736. doi: 10.1371/journal.pone.0188736 (PMC5728480; doi:10.1371/journal.pone.0188736)
Supplement: S1 Text — (DOCX) [file pone.0188736.s001.docx]

**S1 text. Detailed information on the process of neighbourhoods’ classification.**

The socioeconomic characterisation of neighbourhoods was performed using aggregated data from the 2001 National Census regarding Porto city. Statistics Portugal provide authorization to access the database and the unit of observation was the census block, broadly equivalent to a city block in an urban setting [[37](#_ENREF_37)]. The city of Porto area has been divided into 2064 census blocks [[38](#_ENREF_38)], from which 1662 constituted the operational definition for neighbourhoods. Census blocks in which 10 or less individuals lived at the time of the census evaluation were excluded (n=402).

A set of variables related to buildings, households, families and individuals was used to classify each neighbourhood, and most of these variables were provided as absolute counts for each neighbourhood (e.g.: number of women aged 65 years or more, number of persons with 4 complete years of education). To allow comparison between neighbourhoods, we calculated proportions for the variables to be included in the statistical analysis. For example, to create the indicator “proportion of residents with higher education”, we divided the absolute number of residents with university degree or higher by the absolute number of residents. Using this information, we assembled the largest possible group of indicators thought to plausibly represent different socioeconomic aspects of neighbourhoods.

Forty seven indicators were considered in the initial database, but after a descriptive analysis, we noted that some indicators had important floor or ceiling effects. By limiting heterogeneity, the asymmetries in the distribution of these indicators limited their contribution to the characterisation of socioeconomic inequalities. Thus, 15 indicators for which 20% or more of the neighbourhoods presented the minimum or maximum possible value were excluded. Then, after observing correlations ≥0.8 within three subsets of the 32 remaining variables (subset 1: 4 variables, subset 2: 3 variables and subset 3: 2 variables), only one variable from each of the subsets was selected. This procedure led to the exclusion of 6 variables. After evaluating the remaining dataset, there was enough information to characterise three distinct socioeconomic dimensions: age composition of residents within neighbourhoods, education/occupation and housing characteristics. Three to four variables were chosen for each dimension in order to ensure a minimum reliability of 70% and to avoid overrepresentation of specific socioeconomic dimensions. The selection of these variables was first performed independently by three investigators and then decided by consensus, resulting in the exclusion of 15 variables. Finally, the dataset comprised the following 11 variables: proportion of retired individuals, proportion of families with a person aged 15 years or less, aging index, illiteracy proportion, proportion of subjects with higher education, proportion of subjects with lower occupation, unemployment rate, mean expenditure on housing (owner occupied housing), mean expenditure on housing (rented housing), attractiveness (proportion of residents that resided in another territorial unit or country 5 years before) and proportion of buildings with reparation needs [[39](#_ENREF_39)].
